# REIMAGINING POLICY-INFORMED RESEARCH FOR THE FUTURE OF ASSISTED LIVING

**DOI:** 10.1093/geroni/igac059.1119

**Published:** 2022-12-20

**Authors:** Paula Carder, Kali Thomas, Lindsey Smith, Sheryl Zimmerman

**Affiliations:** Portland State University, Portland, Oregon, United States; Brown University, Providence, Rhode Island, United States; Brown University, Providence, Rhode Island, United States; University of North Carolina, Chapel Hill, North Carolina, United States

## Abstract

Researchers have long known that states are responsible for licensing assisted living (AL). Recent analyses indicate that the regulatory landscape is more complex, as states have numerous license classifications that can be combined in multiple ways through various subtypes and designations applied to settings under the AL umbrella. Other sources of complexity include within-state policy variation in terms of affordability and access, person-directed care, emergency preparedness, workforce, and diversity and inclusion. This presentation describes a vision and framework for future research informed by this complex policy landscape. We describe how researchers can better conceptualize types of ALs, and provide guidance for how and why AL researchers might shift from the current practice of analyzing AL settings by the “state” versus by license type, among other categorizations.

